# A case of Kounis syndrome presenting as coronary artery spasm associated with cefazolin-induced anaphylaxis during general anesthesia

**DOI:** 10.1186/s40981-019-0269-3

**Published:** 2019-07-31

**Authors:** Masami Sato, Toshiyuki Arai

**Affiliations:** Department of Anesthesia, Kyoto City Hospital, 1-2 Mibuhigashitakada-cho, Nakagyo-ku, Kyoto, 604-8845 Japan

**Keywords:** Kounis syndrome, Coronary artery spasm, Anaphylaxis, General anesthesia, Cefazolin

## Abstract

**Background:**

Kounis syndrome (KS) is defined as the occurrence of acute coronary syndrome (ACS) associated with an anaphylactic reaction, and there have only been a few reports of its occurrence under general anesthesia.

**Case presentation:**

A 69-year-old woman underwent transurethral resection of a bladder tumor under general anesthesia. Cefazolin was administered intravenously after induction of general anesthesia. During the operation, we suspected ACS from sudden ST segment depression on electrocardiogram. The delayed onset of an erythematous rash reminded us of the anaphylactic reaction of KS. Coronary artery spasm of type 1 KS was diagnosed based upon the findings of coronary computerized tomography. Eleven days after the first surgery, the patient underwent nephroureterectomy uneventfully by a change in antibiotics. Finally, cefazolin proved to be the trigger drug by the intradermal test.

**Conclusion:**

When electrocardiogram changes suggesting ACS occur during general anesthesia, it is necessary to take KS into consideration as a differential diagnosis.

## Background

Kounis syndrome (KS), also known as allergic angina, has been described as an acute coronary syndrome (ACS) associated with an anaphylactic reaction. In KS, a spastic contraction of coronary smooth muscle cells that could progress to acute myocardial damage or immediate coronary thrombosis is caused by the release of inflammatory mediators from mast cells during the allergic activation process [1]. After the first detailed report of the allergic angina syndrome as KS in 1991 [2], recognition of KS has increased recently in emergency and cardiovascular medicine [3, 4]. On the other hand, in the field of anesthesia, this syndrome is considered to be underdiagnosed, and to date, there have only been a few reports describing its occurrence and management during general anesthesia (GA).

Herein, we describe a patient who showed ST segment depression on electrocardiogram (ECG) and hypotension that suggested ACS and was subsequently diagnosed as KS after the appearance of a cefazolin-induced anaphylactic erythematous rash under GA.

## Case presentation

A 69-year-old woman (141 cm, 45 kg) was scheduled to undergo transurethral resection of bladder tumor (TUR-BT) and laparoscopic left nephroureterectomy under the diagnosis of bladder cancer and ureteral cancer. She was treated with trichlormethiazide for hypertension and did not have any history of allergy or ACS. Preoperative ECG showed a flat ST segment. Routine laboratory investigations were normal. Within the past year, the patient had undergone TUR-BT twice under spinal anesthesia and once under GA using propofol, desflurane, fentanyl, remifentanil, and rocuronium. Cefazolin was administered intravenously during every surgery.

After placement of a thoracic epidural catheter, GA was induced with propofol, rocuronium, and fentanyl, and it was maintained with desflurane, remifentanil, and rocuronium. After the trachea was intubated, cefazolin was administered intravenously and TUR-BT started. Forty-five minutes after induction of GA, the resistance of the respiratory tract increased gradually under sufficient effect of rocuronium without any mechanical respiratory problem. Tidal volume in the mechanical ventilator decreased approximately from 350 to 250 ml under pressure-controlled ventilation. Percutaneous oxygen saturation (SpO_2_) decreased from 100 to 94% with a fraction of inspired oxygen of 40% without wheeze. Sixty minutes after induction of GA, the ST segment in lead II of the ECG decreased to − 1.0 mV and isosorbide dinitrate was administered intermittently under the suspicion of ACS. Because of further progression of ST segment depression, we initiated continuous intravenous infusion of nitroglycerin and nicorandil. As the systolic blood pressure (SBP) dropped to 70 mmHg, continuous phenylephrine and intermittent ephedrine were administered to maintain SBP at 80 mmHg. Seventy-three minutes after induction of GA, TUR-BT was finished and the ST segment decreased to − 3.4 mV maximally. When the surgical drape was removed, an anaphylactic erythematous rash was observed on the abdomen, chest, arms, and legs. Then, we suspected anaphylactic reaction to an unknown agent of KS. Corticosteroid and chlorpheniramine were administered immediately.

Transthoracic echocardiography findings were within normal limits. The 12-lead ECG revealed ST depression in I, II, aVF, and V3–6. The laboratory findings for troponin and CK-MB were normal. As the patient responded well to the treatment for anaphylaxis, we were able to stop continuous phenylephrine administration 101 min after induction of GA and the ST depression began to return gradually. Subsequent left nephroureterectomy was postponed and ureteral stent insertion was carried out. When the ureteral stent insertion ended 166 min after induction of GA, blood pressure and the ST depression returned to almost normal with nitroglycerin and nicorandil support. The cutaneous symptoms diminished and SpO_2_ returned to 100% under the same oxygen concentration. Rocuronium was reversed by sugamadex and the trachea was extubated. The vital signs were stable and the patient was free of chest pain and respiratory discomfort. She was transferred to the ICU in order to observe any recurrence of anaphylactic reaction and stayed overnight uneventfully. Based upon the findings of normal coronary vasculature in coronary computerized tomography (CT) 4 days after TUR-BT, the cardiologist diagnosed the case as type 1 variant KS. Five days after TUR-BT, intradermal tests with all of the drugs used during GA were negative.

Eleven days after TUR-BT, laparoscopic left nephroureterectomy was performed under GA and epidural anesthesia. Since cefazolin was one of suspected causes of KS at that time, ciprofloxacin was administered in the ward before surgery and no reaction was observed. Preventative intravenous corticosteroid and chlorpheniramine were administered before GA, and continuous intravenous nicorandil was administered during surgery. GA was induced by thiamylal and suxamethonium and maintained with desflurane, fentanyl, and remifentanil. The minimum dose of rocuronium was administered intermittently to keep T1 of train-of-four under neuromuscular monitoring throughout the surgery. The surgery was completed uneventfully. The patient was discharged on the 18th day after the first operation. The intradermal test performed 8 months later proved that cefazolin was a trigger drug.

## Discussion

KS has three variants [1, 5]; type 1 includes patients with normal coronary arteries in whom coronary artery spasm occurs as a result of the release of inflammatory mediators during an acute anaphylactic reaction. Patients with preexisting atheromatous disease in whom an anaphylactic reaction induces plaque erosion or rupture leading to ACS comprise the type 2 variant. Type 3 KS involves patients with coronary artery stent thrombosis. Since the present case exhibited an episode of ST depression on ECG that was diagnosed as coronary vasospasm based upon coronary CT, cardiac echography, and laboratory investigation, she proved to be the type 1 variant KS.

KS might result in severe conditions, including death, usually as a consequence of myocardial infarction [4, 6]. Therapeutic management of KS needs to treat both cardiac and anaphylactic symptoms simultaneously [3, 7]. However, the drugs to treat KS should be chosen carefully. The use of morphine, which is administered in ACS, can be detrimental due to its histamine-releasing property in KS. The administration of epinephrine to treat anaphylactic reaction can be hazardous due to its alpha-receptor-mediated coronary vasoconstriction, so it should be reserved only for severe cases [7]. With respect to type 1 KS, which is the most common type of KS, vasospasm can be reversed easily by coronary vasodilators, steroids, and anti-histamine drugs, and a good prognosis is expected with appropriate treatment [4]. The present case of type 1 KS also recovered smoothly in response to treatment, and epinephrine was not administered due to the concern of aggravation of coronary artery spasm and stability of the vital signs.

Several causes have been reported to induce KS, including drugs, food, environmental exposures, and drug-eluting stents. The case reports of KS under GA have described rocuronium [8], cisatracurium [9], protamine [10], and diclofenac [11] as suspected triggers. A skin test to determine the trigger drug of anaphylaxis should be performed 4–6 weeks after the anaphylactic reaction, in order to allow recovery of allergic mediators from mast cells and basophils [12]. In our case, the first skin test was performed 5 days after TUR-BT in order to allow early operation to remove ureteral cancer. The reason why the responsible allergen was not identified was the suppression of the immune response, judging from the fact that the skin test response to histamine was also negative.

It has been reported that antibiotics are the most frequently involved drugs in KS and that rocuronium is associated with a high incidence of anaphylaxis [6, 13]. Thus, we estimated that cefazolin or rocuronium were more likely to be the possible anaphylaxis-inducing drugs before the second surgery. In the second surgery, a different antibiotic from cefazolin was administered preoperatively to the monitored awake patient. As 40% of patients diagnosed with rocuronium anaphylaxis have been reported to exhibit cross-reactivity to vecuronium, another available non-depolarizing neuromuscular agent in Japan [13], we chose to use rocuronium carefully again. After all, cefazolin proved to be the trigger of KS. Given that the patient had been exposed to cefazolin three times prior to the initial surgery, cefazolin would have sensitized the patient.

The time from exposure to trigger to the onset of KS has been reported to be within 1 h in 80% of KS patients [4]. As many drugs, including anesthetics, neuromuscular blocking drugs, and antibiotics, are administered during GA, identification of the anaphylaxis-inducing trigger drug is difficult in rapid succession, especially during induction of GA. The clinical manifestations of KS in anesthetized patients who do not exhibit subjective symptoms often differ from those who are awake. Among clinical signs of anaphylaxis, respiratory manifestations are less common during GA. Moreover, cutaneous symptoms are not always manifested and can be difficult to detect because of surgical drapes. Initial symptoms of KS under GA have been reported to be ECG changes or hypotension in 3 of 4 previous reports [8, 10, 11], and respiratory symptoms were only reported in one of these reports [9]. In our case, the decreased SpO_2_ without any special causes 45 min after induction of GA, which might have been initial symptom of KS, did not immediately remind us of anaphylaxis.

When ECG changes suggestive of ACS occur during GA, it is important to monitor the respiratory and cutaneous symptoms carefully, in consideration of anaphylactic manifestation of KS as a differential diagnosis.

## Data Availability

The data are not available for public access because of patient privacy concerns, but are available from the corresponding author on reasonable request.

## Abbreviations

ACS: Acute coronary syndrome
CT: Computerized tomography
ECG: Electrocardiogram
GA: General anesthesia
KS: Kounis syndrome
SBP: Systolic blood pressure
SpO_2_: Percutaneous oxygen saturation
TUR-BT: Transurethral resection of bladder tumor
